# Role of tyramine in calcium dynamics of GABAergic neurons and escape behavior in *Caenorhabditis elegans*

**DOI:** 10.1186/s40851-018-0103-1

**Published:** 2018-07-26

**Authors:** Yuko Kagawa-Nagamura, Keiko Gengyo-Ando, Masamichi Ohkura, Junichi Nakai

**Affiliations:** 1 Brain and Body System Science Institute, Saitama University, 255 Shimo-okubo, Sakura-ku, Saitama, 338-8570 Japan; 2 Graduate School of Science and Engineering, Saitama University, 255 Shimo-okubo, Sakura-ku, Saitama, 338-8570 Japan; 3 RIKEN Center for Brain Science, 2-1 Hirosawa, Wako-shi, Saitama, 351-0198 Japan

**Keywords:** *Caenorhabditis elegans*, Tyramine, Calcium imaging, Neuromodulation, Escape behavior

## Abstract

**Background:**

Tyramine, known as a “trace amine” in mammals, modulates a wide range of behavior in invertebrates; however, the underlying cellular and circuit mechanisms are not well understood. In the nematode *Caenorhabditis elegans* (*C. elegans*), tyramine affects key behaviors, including foraging, feeding, and escape responses. The touch-evoked backward escape response is often coupled with a sharp omega turn that allows the animal to navigate away in the opposite direction. Previous studies have showed that a metabotropic tyramine receptor, SER-2, in GABAergic body motor neurons controls deep body bending in omega turns. In this study, we focused on the role of tyramine in GABAergic head motor neurons. Our goal is to understand the mechanism by which tyraminergic signaling alters neural circuit activity to control escape behavior.

**Results:**

Using calcium imaging in freely moving *C. elegans*, we found that GABAergic RME motor neurons in the head had high calcium levels during forward locomotion but low calcium levels during spontaneous and evoked backward locomotion. This calcium decrease was also observed during the omega turn. Mutant analyses showed that *tbh-1* mutants lacking only octopamine had normal calcium responses, whereas *tdc-1* mutants lacking both tyramine and octopamine did not exhibit the calcium decrease in RME. This neuromodulation was mediated by SER-2. Moreover, tyraminergic RIM neuron activity was negatively correlated with RME activity in the directional switch from forward to backward locomotion. These results indicate that tyramine released from RIM inhibits RME via SER-2 signaling. The omega turn is initiated by a sharp head bend when the animal reinitiates forward movement. Interestingly, *ser-2* mutants exhibited shallow head bends and often failed to execute deep-angle omega turns. The behavioral defect and the abnormal calcium response in *ser-2* mutants could be rescued by SER-2 expression in RME. These results suggest that tyraminergic inhibition of RME is involved in the control of omega turns.

**Conclusion:**

We demonstrate that endogenous tyramine downregulates calcium levels in GABAergic RME motor neurons in the head via the tyramine receptor SER-2 during backward locomotion and omega turns. Our data suggest that this neuromodulation allows deep head bending during omega turns and plays a role in the escape behavior in *C. elegans*.

**Electronic supplementary material:**

The online version of this article (10.1186/s40851-018-0103-1) contains supplementary material, which is available to authorized users.

## Background

Neuromodulators play important roles in the control of behavior by modifying neural network function [1, 2]. Biogenic amines such as serotonin, dopamine, and noradrenaline are major neuromodulators that affect many physiological and behavioral processes. In invertebrates, noradrenaline/adrenaline are not biosynthesized, but structurally related octopamine and its precursor tyramine are often considered as the invertebrate counterparts of the vertebrate adrenergic transmitters [3]. The tyraminergic/octopaminergic system has been studied extensively in insects and nematodes, and has been shown to affect many neural functions, including locomotion [4], arousal [5, 6], starvation response [7, 8], learning and memory [9, 10], decision-making [11], and the fight or flight response [12]. However, the underlying cellular and circuit mechanisms are not well understood. In mammals, tyramine and octopamine are detected as “trace amines” in the brain and nervous tissues, and their physiological roles are largely unknown [13]. The recent discovery of the mammalian G-protein-coupled receptor with high affinity for tyramine, TAAR1 [14], and the implications of TAAR1 in neurological disorders [15] highlight the potential importance of tyraminergic signaling in vertebrate nervous systems.

*Caenorhabditis elegans* (*C. elegans*) is a useful model for studying monoamine function and modulation of behaviors. In wild-type worms, the tyramine concentration is 0.3 ± 0.1 pmol/mg of wet weight, detected using high-performance liquid chromatography (HPLC) [16]. The *C. elegans* nervous system has only 302 neurons, which form approximately 7000 chemical synapses and 600 gap junctions [17, 18]. Tyramine is biosynthesized in a pair of ring inter neuron/motor (RIM) neurons and controls key behaviors including foraging, pharyngeal pumping, and escape responses [16, 19]. A gentle touch to the head of the animal induces a rapid reversal and suppression of foraging. Long reversals are often coupled with a subsequent sharp ventral turn called an “omega turn” enabling animals to navigate away in the opposite direction. This complex motor sequence is coordinated by the tyraminergic modulation of neurons and muscles through the synaptic or extrasynaptic activation of different tyramine receptors [20]. In omega turns, the extrasynaptic activation of the metabotropic tyramine receptor SER-2 modulates GABAergic body motor neurons to enable deep body bending [20]. In the head motor circuit, however, the role of tyramine during omega turns remains unknown.

To address this issue, we focused on the role of tyramine in GABAergic head motor neurons, ring motor neuron class E (RME) neurons that regulate head movement. Our goal is to understand the mechanism by which tyraminergic signaling alters neural circuit activity to control escape behavior. Recent advances in fluorescent calcium indicators [21, 22] and optogenetic tools [23] have facilitated the study of neural dynamics in vivo, particularly in transparent organisms such as *C. elegans* [24, 25]. *C. elegans* lacks voltage-dependent sodium channels, but has genes encoding voltage-dependent calcium channels in its genome [26]. In fact, the intracellular calcium concentration in *C. elegans* neurons changes dynamically, and calcium measurements have often been used for the assessment of neural activity and function in this model. Our preliminary calcium imaging data have suggested that RME are affected by tyramine (Gengyo-Ando et al., 2013; the 19th international worm meeting, University of California, Los Angeles). In the present study, to further understand the neuromodulation of RME, we examined the correlation between the calcium dynamics of RME and behavior using an autotracking imaging system, ICaST [27]. Our data demonstrate that tyramine alters calcium levels in RME neurons in a specific behavioral state. Combining genetic mutant analysis, behavioral analysis, and cell-specific rescue experiments, we elucidated the role of tyraminergic signaling in omega turns.

## Methods

### *C. elegans* strains and general methods

*C. elegans* strains were maintained at 20 °C on nematode growth media (NGM) with *Escherichia coli* OP50–1 as a food source as per standard methods [28]. The strains QJ4112, QJ4119, QJ4122, and QJ4123 (Additional file 1: Table S1) were cultured at 15 °C because they exhibited partial sterility at 20 °C. Transgenic strains carrying extrachromosomal DNA arrays were generated using the standard microinjection method [29]. Chromosomally integrated DNA arrays were generated using the UV irradiation method [30]. *Punc-122::mCherry* or *Punc-122::EYFP* was used for injection markers. The transgenic strains generated in this study are listed in Additional file 1: Table S2. Transgenic strains harboring mutant alleles were created by genetic crosses or direct injection of transgenes into mutant animals. Genotyping of the deletion alleles *tdc-1 (ok914)*, *tbh-1 (ok1196)*, *ser-2 (ok2103)*, *tph-1 (mg280)*, *lgc-55 (n4331)*, *tyra-3 (ok325)*, and *cat-2 (jq6)* were performed by polymerase chain reaction (PCR). Primers used for genotyping are listed in Additional file 1: Table S3.

### Molecular biology

cDNA of G-CaMP7 [31] and R-CaMP2 [32] were amplified by PCR from the original plasmids. A codon-optimized G-CaMP7 for *C. elegans* (G-CaMP7ce) was de novo synthesized. The 1.3 kb full-length *ser-2* cDNA (*ser-2 (+)*) was amplified by reverse transcription-PCR (RT-PCR) from the N2 poly A (+) RNA. Each cDNA sequence was cloned into a pPD49.26 [33] or a pFX_EGPT vector [34]. The following promoters were amplified by PCR from the *C. elegans* N2 genomic DNA and were inserted upstream of cDNA sequences (expressing cells are shown in parenthesis): 2.8 kb *tbh-1* (RIC, RIM) [16]; 4.4 kb *tdc-1* (RIM) [16]; 1.9 kb *unc-25* [35] or 1.2 kb *unc-47* [36] (GABAergic neurons); 3.2 kb *unc-17* (cholinergic neurons) [37]; 2.4 kb *unc-30* (D-type GABAergic neurons) [38]; and 4.7 kb *ser-2prom2* (RME and subsets of non-GABAergic neurons) [39]. The amplified cDNAs were verified by DNA sequence analysis. Primers used for PCR and sequence analysis are listed in Additional file 1: Table S4.

### Calcium imaging and optogenetics

Calcium imaging of RME and RIM in freely behaving animals was performed using the autotracking imaging system ICaST as described previously (Additional file 1: Figure S1) [27]. Briefly, to automatically track a moving animal in the field of view of the laser-scanning microscope, infrared worm images were taken at 200 frames/s and image processing was conducted in real-time. G-CaMP7 and green fluorescent protein (GFP) were excited by a 488 nm laser, and R-CaMP2 and red fluorescent protein DsRed were excited by a 561 nm laser. Blue fluorescent protein EBFP was excited by a 405 nm laser. Fluorescence and transmitted light images (512 × 512 pixels/image) were captured at 7~ 15 fps with a 20× objective (CFI Plan Apo λ, NA0.75, Nikon) or a 25× objective (Plan Apo LWD, 25× NA1.10, Nikon). Animals were placed between a coverslip and 1.0%~ 1.5% agar pad on a glass slide.

For optogenetic analysis, transgenic animals expressing channelrhodopsin-2 (ChR2) in ASH neurons were cultivated on NGM with all-*trans*-retinal (ATR) (Sigma-Aldrich Japan, KK, Tokyo) in the dark for 1–3 days before experiments. Simultaneous imaging and optogenetic control was performed as described previously [27]. To avoid inappropriate excitation of G-CaMP, blue light at 440 ± 10.5 nm, which is far from the excitation peak of G-CaMP7, was used for the photoactivation of ChR2. To minimize optical crosstalk, the intensities of the 488 nm laser and 440 nm blue light were optimized. Detailed methods for controlling the light intensity are described in a previous report [27]. For calcium imaging of optogenetically evoked reversals and omega turns, photostimulation for 5–20 s was delivered after animals moved forward for at least 5 s.

### Analysis of imaging data

Imaging data were analyzed using the NIS-elements AR software (ver.3.22, Nikon, Tokyo) as described previously [27]. Briefly, in the case of transgenic animals expressing G-CaMP7 and DsRed, each ROI of a neuron was tracked offline using DsRed signals, and then the G-CaMP7 and DsRed fluorescence intensities were analyzed. The fluorescence intensity ratio (*R*) was calculated as G-CaMP7/DsRed. The ratio change in reversals was calculated as *R*_*backward*_/*R*_*forward*_ × 100%, where *R*_*backward*_ is the mean *R* during backward locomotion lasting more than 1.5 s and *R*_*forward*_ is the mean *R* during forward locomotion for 3 s (*t* = − 4 s to − 1 s). Imaging data for 2 s (*t* = − 1 s to 1 s) were excluded from the data analysis because this period corresponds to the rising or falling phase of calcium signals. The time point of the directional switch (*t* = 0) in the forward to backward locomotion was detected by visual inspection of the transmitted light images. The ratio change in the omega turn was calculated as *R*_*omega turn*_ / *R*_*forward*_ × 100%, where *R*_*omega turn*_ is the mean *R* during the initial phase (1 s) of omega turn immediately after the reversal phase, and *R*_*forward*_ is the mean *R* during forward locomotion (1 s) from − 5 s to − 4 s before photostimulation (Fig. 5f).

### 3D imaging of RIM and RME neurons

Z-stacks with 0.5 μm spacing were taken using a confocal laser microscope A1R (Nikon) with a 60× objective (Plan Apo λ, 60× NA1.40, Nikon), and 3D images were reconstituted with the maximum intensity projection program of NIS-element (Nikon). For imaging, 405, 488, and 561 nm lasers were used.

### Omega turn assays

Omega turn assays were performed on foodless NGM assay plates (60 mm diameter) as previously described [40]. Five well-fed young adult animals were transferred to the assay plate and tested 1 min after the transfer. Omega turns were induced by anterior touches with an eyelash. At most five touches were performed per animal, and the first omega turn was scored under the observation of a stereo-microscope (SZX16, Olympus, Tokyo). The omega turn was defined as a sharp turn of which the turning angle was greater than 120° after a reversal with three or more body bends. A normal omega turn was defined as a turn in which the head touched the tail. For each strain, 100 animals were scored (Fig. 5h). For analyzing the head bending angle, omega turns were recorded using a digital microscope camera (Ds-Fi1, Nikon, Tokyo) mounted on the stereo-microscope and the NIS-Elements BR software ver 4.11 (Nikon, Tokyo). Images of 640 × 480 pixel sizes were captured at 12 frames/s. One frame immediately before the initiation of an omega turn was extracted and analyzed. A midline of the worm image was equally divided into 12 segments using software wriggle tracker version 1.2 and move-tr/2D software ver. 8.31 (Library Co., Ltd., Tokyo, Japan). The tip of the head was numbered as point 1, and the end of the tail was numbered as point 13 (Fig. 5d). The bending angle formed by three points (point 1, 2, and 3) was measured and analyzed for each image (Fig. 5e).

### Correlation analysis

The correlation coefficient of calcium dynamics between RME pairs was calculated using Excel (Microsoft). Cross-correlation analysis between RME and RIM activities (Fig. 3f) was conducted using the function ccf in the free statistical programming language R (www.r-project.org) after processing with moving average (0.27 s window) followed by normalization of the fluorescent intensity ratio values.

### Statistical analysis

**Fig. 1:**
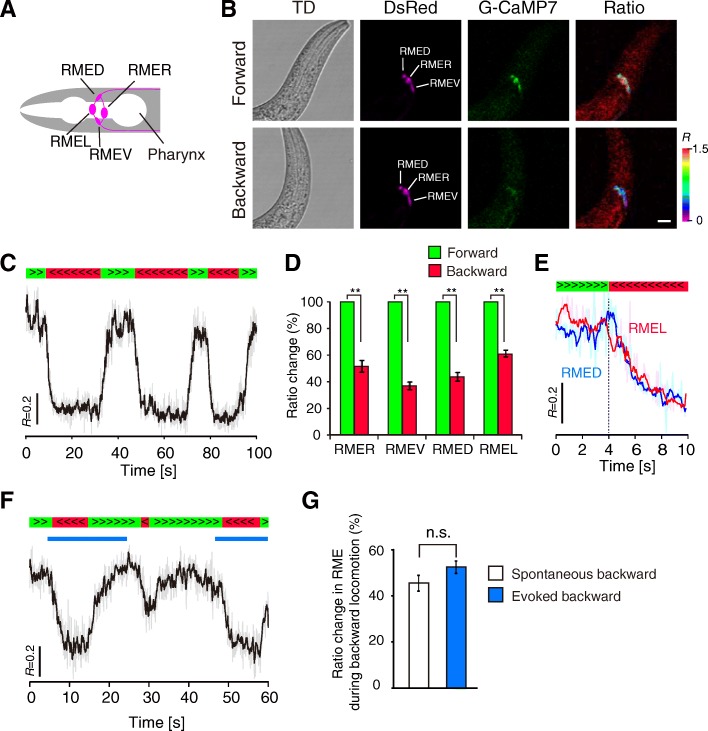
RME neurons are synchronously inhibited during backward locomotion. **a** Schematic of RME. **b** Representative fluorescent and transmitted-light microscopy images of a transgenic animal (*jqIs72*) expressing G-CaMP7 and DsRed during forward movement (Top) and backward movement (Bottom). Pseudocolor images show the fluorescence intensity ratio (*R* = G-CaMP7/DsRed). Scale bar represents 10 μm. **c** A representative calcium trace of RME during spontaneous locomotion. The gray line shows raw data, and the black line shows the moving average data across a 0.27 s window. Periods of forward locomotion (green bars) and backward locomotion (red bars) are indicated on the top of the trace. **d** Quantitative analysis of fluorescence ratio changes from each RME cell type. *n* = 8–10 from 6 to 9 animals. **P < 0.01, Student’s two-tailed t-test. Error bars, SEM. **e** Typical calcium dynamics of RMEL (red) and RMED (blue) associated with the directional change from forward (green bar) to backward (red bar) locomotion. In both neurons, calcium decrease begins at the onset of reversal and persists during backward movement. Light colors show raw data, and dark colors show moving averages of ratio values (*R*). **f** ChR2-mediated photostimulation (blue bars) of ASH and simultaneous calcium imaging of RME. **g** Mean fluorescence ratio changes during the optogenetically induced reversals (*n* = 12 from seven animals) were comparable with those during spontaneous reversals (*n* = 14 from six animals). *n.s.* = not significant, Student’s two-tailed t-test. Error bars, SEM

**Fig. 2:**
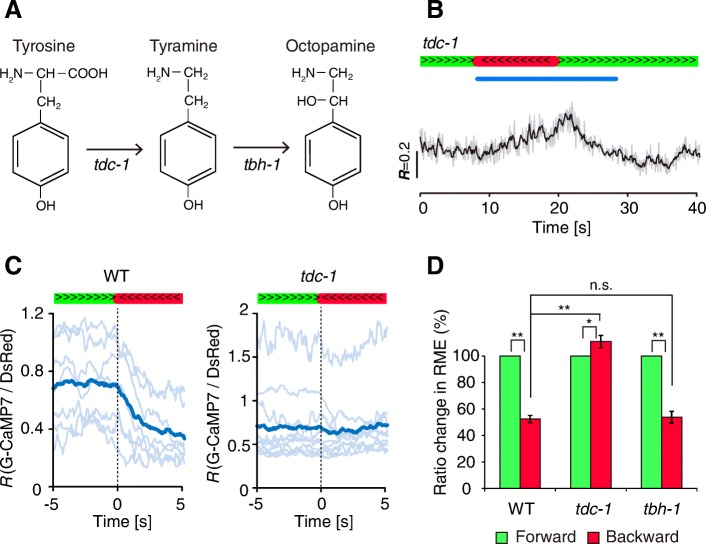
Tyramine downregulates calcium activity in RME. **a** Biosynthetic pathway of tyramine and octopamine. Genes encoding synthetic enzymes are shown under the arrows. **b** Calcium dynamics of RME in the *tdc-1(ok914)* mutant. The gray line shows raw data, and the black line shows moving average data of fluorescence intensity ratio (*R* = G-CaMP7/DsRed). Green bar: forward locomotion; red bar: backward locomotion; blue bar: photostimulation. **c** Calcium dynamics of directional switching (*t* = 0) from forward (green) to backward (red) movements from WT (*n* = 7 from five animals) and *tdc-1 (ok914)* (*n* = 10 from seven animals). Light-blue lines show raw data from individual animals, and dark-blue lines show mean values. Green bar: forward locomotion; red bar: backward locomotion. **d** Quantitative analysis of fluorescence ratio changes of RME in WT, *tdc-1 (ok914)* and *tbh-1 (ok1196)*. *n* = 7–10 from 5 to 7 animals. **P < 0.01, *P < 0.05, *n.s.* = not significant, Student’s two-tailed t-test and one-way ANOVA with Tukey’s test. Error bars, SEM

**Fig. 3:**
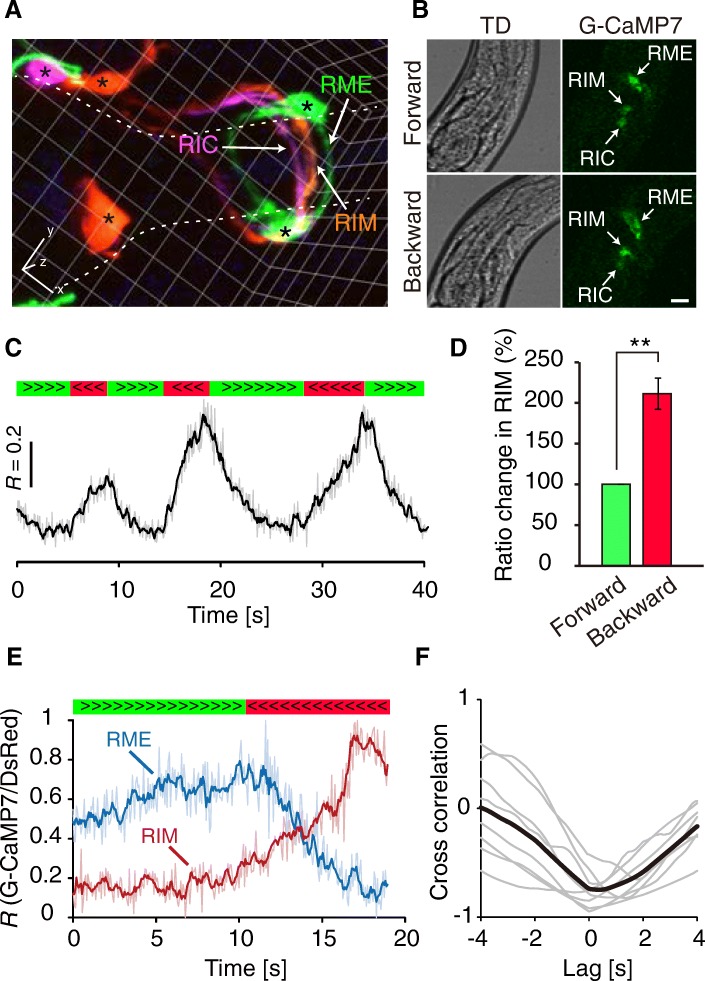
Tyraminergic RIM neurons are activated during backward locomotion. **a** 3D reconstructed fluorescent image of the head region of a *jqIs75;jqEx516* worm*.* RIM (orange), RIC (pink), and RME (green) are shown. Cell bodies are indicated by asterisks. Dashed lines denote the pharynx. Graticule size is 5 μm. **b** G-CaMP images and the corresponding transmitted-light images of a transgenic animal (*jqIs73;jqIs75*) expressing G-CaMP7 in RIM and RME. Scale bar represents 10 μm. **c** Representative calcium trace of RIM. The light-gray line shows raw data of ratio (*R*: G-CaMP7 / DsRed), and the black line shows the moving average of *R* across a 0.27 s window. Periods of forward and backward locomotion are indicated by the green and red bars, respectively. **d** Quantitative analysis of the fluorescence ratio changes of RIM (*n* = 9 from six animals). **P < 0.01, Student’s two-tailed t-test. Error bars, SEM. **e** Calcium dynamics of RIM (red) and RME (blue) recorded from an animal. Light colored lines show normalized raw data, and dark colored lines show moving averages across a 0.27 s window. RIM and RME show reciprocal calcium transients in the directional switch from forward (green bar) to backward (red bar) locomotion. **f** Cross-correlation analysis of calcium dynamics between RIM and RME. Gray lines are individual data (*n* = 8), and the black line shows a mean trace

**Fig. 4:**
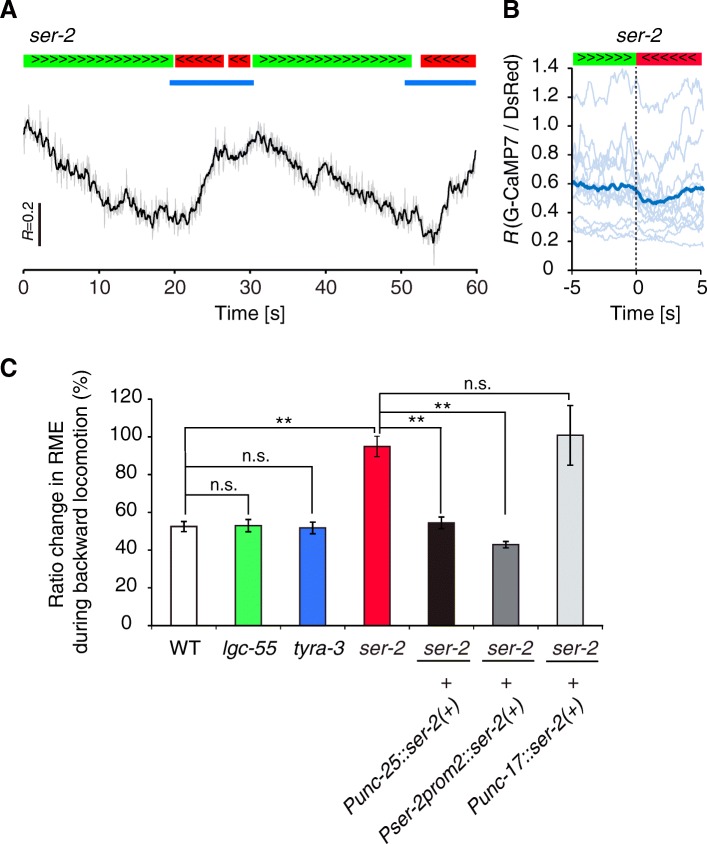
RME calcium activity is regulated by SER-2. **a** Representative calcium trace of RME in the *ser-2 (ok2103)* mutant. The gray line shows the fluorescence ratio value (*R*: G-CaMP/DsRed) and the black line shows moving averages of *R*. Periods of forward (green bars) and backward (red bars) locomotion and photostimulation (blue bars) are shown. **b** Calcium dynamics of directional switching (*t* = 0) from forward (green bar) to backward (red bar) movements. Light-blue lines show raw data from individual animals, and the dark-blue line shows mean values (*n* = 15 from six animals). **c** Rescue experiments of the *ser-2* mutant. Mean fluorescence ratio changes of RME during backward movement were quantitatively analyzed in tyramine receptor mutants and *ser-2* rescue transgenic lines. WT data were derived from Fig. 1g (*n* = 8–14 from 5 to 7 animals). **P < 0.01, *P < 0.05, *n.s.* = not significant, one-way ANOVA with Tukey’s test. Error bars, SEM

**Fig. 5:**
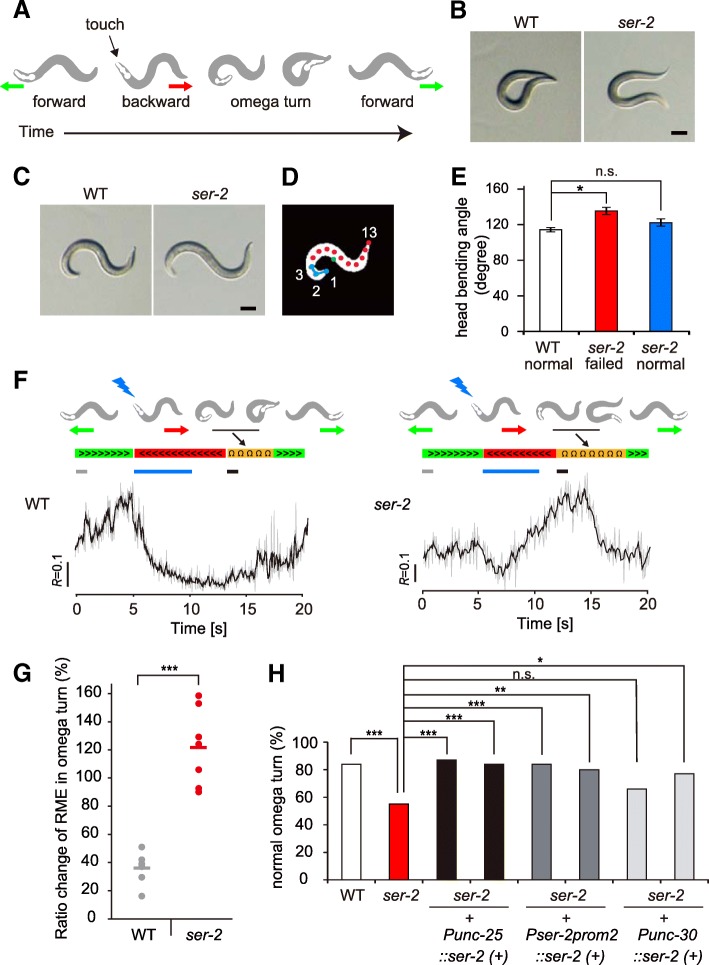
SER-2 signaling in RME is involved in omega turn behavior. **a** An anterior touch induces a backward escape response accompanied by an omega turn. **b** Representative images of omega turns in WT and *ser-2* mutant worms. A WT animal deeply bends and touches its head to the tail (closed omega turn), but the *ser-2 (ok2103)* mutant often exhibits shallow bends and does not touch its head to the tail. Scale bar represents 100 μm. **c** Representative images of animals initiating omega turns. **d** Schematic of omega turn analysis. The worm’s body was divided into 12 equal segments and the head-bending angle formed by three points (1–2-3) was calculated. The centroid is indicated by a green circle. **e** Quantitative analysis of mean head-bending angle. *Ser-2 (ok2103)* mutant animals exhibit shallow head bends when they failed to close omega turns. *n* = 17 each. **P < 0.01, *P < 0.05, one-way ANOVA with Tukey’s test. Error bars, SEM. **f** Calcium dynamics of RME during optogenetically evoked escape behavior. Periods of forward movement, backward movement, and omega turns are indicated by green, red, and orange bars, respectively. Illumination with blue light is indicated by blue bars. **g** Quantitative analysis of the fluorescence ratio changes of RME in the initial phase of an omega turn from WT (gray) and *ser-2 (ok2103)* (red) worms. To calculate the fluorescence ratio changes of an omega turn, imaging data for 1 s periods in the forward movement (gray bar) and in the initial phase of the omega turn (black bar) in panel F were used. Individual data and averaged data of fluorescence ratio change are indicated by circles and lines, respectively. ***P < 0.001, Student’s two-tailed t-test. **h** Transgenic rescue experiment of the omega turn phenotype in the *ser-2 (ok2103)* mutant. The success rate of omega turns was evaluated in each strain (*n* = 100). A normal omega turn was defined as an omega turn in which the tip of the nose touches the tail. ***P < 0.001, **P < 0.01, *P < 0.05, *n.s.* = not significant, Chi-squared test and Bonferroni test

Statistical analysis was performed using Ekuseru-Toukei 2010 (version 1.10, Social Survey Research Information Co., Ltd.) and language R. Student’s t-test was used to determine the significance of data in Figs. 1d, g, 3d, 5g, and Additional 1: Figure S2C. One-way ANOVA with Tukey’s test was used for multiple comparisons in Figs. 2d, 4c and 5e. Chi-squared test and Holm correction were used for statistical analysis in Fig. 5h.

## Results

### Calcium activity in RME neurons is synchronously decreased during backward movement

In *C. elegans*, the head and body movements are independently regulated by distinct motor circuits composed of different classes of motor neurons and muscles. The head motor circuit contains several groups of excitatory cholinergic motor neurons and one group of inhibitory GABAergic motor neurons, RME neurons. The muscle contraction is regulated by the balance of direct inputs from these cholinergic and GABAergic motor neurons. In the GABAergic system, 4 RME neurons and 19 D-type motor neurons [6 dorsal D-type (DD) neurons for dorsal muscles and 13 ventral D-type (VD) neurons for ventral muscles] innervate head and body muscles, respectively [18]. The 4 RME neurons are arranged around the nerve ring in the head (Fig. 1a) and RME dorsal (RMED), RME ventral (RMEV), RME left (RMEL), and RME right (RMER) innervate the contralateral muscles in the ventral, dorsal, right, and left muscle quadrants, respectively.

To record calcium responses from RME during behavior, we used the ICaST system, which combines a fast-scanning laser microscope and an automatic worm tracking system (Additional file 1: Figure S1). We generated chromosomally integrated transgenic lines (*jqIs6*, *jqIs71*, and *jqIs72*) expressing both the GFP-based genetically encoded calcium indicator (GECI) G-CaMP7 [31] and the calcium-insensitive red fluorescent protein DsRed in GABAergic neurons (*Punc-25::G-CaMP7*) (Fig. 1b). The transgenic animals were placed between a flat agar pad and a coverslip such that they could freely move on the x–y plane. We automatically tracked the head region containing RME with the texture-tracking algorithm and simultaneously recorded behavior and fluorescent calcium signals. We performed semi-ratiometric measurements by calculating the fluorescent intensity ratio *R* (*R* = G-CaMP7/DsRed) to reduce motion artifacts. Fig. 1c shows a representative calcium trace of RME correlated with spontaneous locomotion. We found that RME showed high calcium levels during forward movement and low calcium levels during backward movement. The calcium decrease began at the onset of backward movement and persisted until the next onset of forward movement. A similar result (Additional file 1: Figure S2) was obtained using a red GECI, R-CaMP2 [32]. Our results are consistent with previous reports that RME neurons are not active in the backward moving states in whole-brain imaging of *C. elegans* [41, 42]. In each RME neuron, the calcium level was significantly decreased during backward movement compared with that during forward movement (RMED 43.6 ± 3.3%, *n* = 9 from seven animals, *P* < 0.01; RMEV 36.9 ± 2.9%, *n* = 10 from nine animals, *p* < 0.01; RMEL 60.8 ± 2.6%, *n* = 9 from seven animals, *P* < 0.01; RMER 51.6 ± 4.4%, *n* = 8 from six animals, *P* < 0.01; mean *R*_*backward*_/*R*_*forward*_ × 100 ± SEM%) (Fig. 1d). We found that the calcium activity was highly correlated between all RME pairs in the directional switch from forward to backward (correlation coefficients *r*: RMED/R 0.79 ± 0.032, *n* = 7; RMEV/R 0.76 ± 0.032, *n* = 8; RMED/L 0.84 ± 0.016, *n* = 10; RMEV/L 0.83 ± 0.022, *n* = 9; RMED/V 0.85 ± 0.019, *n* = 9; RMEL/R were not examined because they were located in the different focal planes) (Fig. 1e). These results suggest that RME neurons are synchronously inhibited in spontaneous backward movement.

We next tested whether the RME calcium decrease is observed in the backward escape response evoked by external stimuli. A pair of amphid sensory neurons with a single ciliated sensory ending, ASHs sense various stimuli to initiate avoidance response. The optogenetic stimulation of ASH using channelrhodopsin-2 (ChR2) can induce the backward escape response [43]. Therefore, we examined the optogenetic control of ASH and the simultaneous readout from RME. To reduce the optical crosstalk between G-CaMP and ChR2, we chose a combination of slightly red-shifted G-CaMP7 and a slightly blue-shifted variant H134R of ChR2 and generated a transgenic line *jqIs6;jqEx318* expressing G-CaMP7 and ChR2 (H134R) in RME and ASH, respectively (*Punc-25::G-CaMP7; Psra-6::ChR2(H134R)*). In addition, we optimized imaging conditions to minimize the optical crosstalk as described previously [27]. When the transgenic animals were illuminated with blue light for optogenetic stimulation, they reliably moved backward in the presence of ATR, an essential cofactor of ChR2 (6 animals responded out of six tested). We found that RME showed a clear reduction in calcium activity during the evoked backward locomotion (Fig. 1f). Quantitative analysis showed that the ratio changes in the evoked reversals (52.5 ± 2.7%, *n* = 14 from six animals) was comparable with that in the spontaneous reversals (45.5 ± 3.4%, *n* = 12 from seven animals) (Fig. 1g). These results indicate that intracellular calcium levels in RME neurons are downregulated in the backward state, irrespective of the presence or absence of sensory signals.

### Tyramine is required for the inhibition of RME

To investigate the neural mechanism of RME inhibition, we analyzed RME activity in mutants of biosynthetic enzymes for biogenic amines. It is known that tyramine, octopamine, serotonin, and dopamine have inhibitory effects on behavior in wild-type *C. elegans* [8, 19, 44]. We first examined genes that encoded biosynthetic enzymes for tyramine and octopamine (Fig. 2a). We found that tyrosine decarboxylase (*tdc-1*) mutants, which are deficient in tyramine and octopamine, did not show any prominent calcium decrease in RME during evoked backward locomotion (Fig. 2b and c). Quantitative analysis showed that calcium levels during backward locomotion were slightly higher than those during forward movement (Fig. 2d) (*tdc-1*, 111.0 ± 4.7%, *n* = 9 from seven animals, *P* < 0.05). This result suggests that tyramine and/or octopamine are required for inhibiting RME. To test this possibility, we examined tyramine beta hydroxylase (*tbh-1*) mutants, which are deficient in only octopamine. Unlike the *tdc-1* mutants, in the *tbh-1* mutant background, the RME calcium level decrease during backward locomotion was comparable with that in the WT background (*tbh-1*, 53.8 ± 4.5%, *n* = 8 from five animals, *P* = 0.97) (Fig. 2d and Additional file 1: Figure S3). These results indicate that tyramine, but not octopamine, has a role in the regulation of calcium decrease in RME. We also examined RME activity in null mutants for tryptophan hydroxylase (*tph-1*) and tyrosine hydroxylase (*cat-2*) genes, which are necessary for serotonin and dopamine synthesis, respectively (Additional file 1: Figure S4). In these mutants, RME had normal calcium responses, suggesting that serotonin and dopamine are not required for the inhibition of RME calcium activity.

### Reciprocal calcium activity between RME and tyraminergic RIM neurons

RIM is the sole tyraminergic neuron class in *C. elegans* [16]. If RME activity is regulated by tyramine, RIM would be active in the backward moving state. In previous studies, both increases and decreases in calcium have been observed in RIM during backward locomotion [45, 46]. To assess RIM activity under our experimental conditions, we performed calcium imaging using a transgenic line, *jqIs75*, that expresses G-CaMP7 in RIM neurons (*Ptdc-1::G-CaMP7*). As expected, in RIM neurons, calcium increase began at the onset of backward movement and persisted until the next onset of forward movement (Fig. 3c). The mean fluorescent ratio change in RIM during backward movement was significantly higher than that during forward movement (Fig. 3d). To further characterize calcium dynamics in RIM and RME at the same time, we generated a transgenic line, *jqIs6;jqIs75*, that expresses G-CaMP7 in these neurons (*Punc-25::G-CaMP7; Ptdc-1::G-CaMP7*) (Fig. 3b). Simultaneous calcium imaging of RIM and RME revealed that in the directional switch from forward to backward, RIM calcium activity increased, whereas RME calcium activity decreased in the same animal (Fig. 3e). Cross-correlation analysis also confirmed that RME activity was negatively correlated with RIM activity (Fig. 3f). The calcium response in RIM slightly proceeded that of RME (lag time 469 ± 264 ms, *n* = 8, *P* < 0.05), suggesting that RME is downstream of RIM in the functional neural network. Although RIM and RME have no direct synaptic connectivity [18], 3D imaging showed that RIM and RME processes were in proximity in the nerve ring (Fig. 3a). Therefore, these results suggest that RME calcium levels are extrasynaptically modulated by tyramine released from RIM during backward movement.

### The tyramine receptor SER-2 is required for calcium decrease in RME

Neuromodulators generally act through metabotropic G-protein-coupled receptors (GPCRs). *C. elegans* has four tyramine receptors: one ionotropic receptor (LGC-55) [47] and three GPCR tyramine receptors (TYRA-2, TYRA-3, and SER-2) [48–50]. Since SER-2 is expressed in a subset of neurons, including GABAergic RME and VD neurons [20, 39], we examined RME calcium transients in *ser-2* deletion mutants (Fig. 4a and b). In *ser-2* mutants, like in *tdc-1* mutants, RME had significantly high calcium levels during backward locomotion (*ser-2*, 94.9 ± 5.4%, *n* = 14 from six animals, *P* < 0.01) (Fig. 4c). We also examined deletion mutants for *tyra-3* [50] and *lgc-55* [47] and found that these mutants had normal calcium response (*tyra-3*, 51.7 ± 3.1%, *n* = 12 from five animals *P* = 1.00; *lgc-55*, 53.0 ± 3.2%, *n* = 13 from six animals *P* = 1.00) (Fig. 4c). These results suggest that SER-2 is required for the tyraminergic inhibition of RME calcium levels.

We next examined whether expression of a wild-type *ser-2 (+)* transgene in RME could rescue the neural defect in *ser-2* mutants. There is currently no specific promoter for expression in only RME. Therefore, we used the *ser-2prom2* promoter [39], which can drive expression in RME and a subset of non-GABAergic neurons. We found that the transgene (*Pser-2prom2::ser-2(+)*) rescued the defect in calcium response in *ser-2* mutants to the same extent as expression of *ser-2 (+)* in all GABAergic neurons (*Punc-25::ser-2(+)*) (Fig. 4c). These two rescue genes show overlapping expression in only RME, suggesting that the SER-2 receptor acts cell-autonomously in RME to modulate calcium levels in the backward moving state. In a control experiment, expression of *ser-2 (+)* in all cholinergic neurons (*Punc-17::ser-2 (+)*) had no rescue activity (Fig. 4c).

### Tyramine affects omega turns by modulating RME neurons through SER-2

A previous report has shown that in the touch-evoked escape response, wild-type animals usually exhibit omega-shaped sharp turns (omega turns) after long reversals, whereas *ser-2* mutants often exhibit shallow omega turns in which the head does not touch the tail [20]. Because the omega turn is initiated by a sharp head bend, we were interested in whether SER-2 signaling in RME affects omega turns. We first tested whether the shallow omega turn in *ser-2* mutants is reproducibly observed under our experimental conditions. We performed anterior touches to animals to induce escape responses and scored normal omega turns in which the head touched the tail (*n* = 100) (Fig. 5a and b). Consistent with the previous report, we observed that wild-type animals exhibited normal omega turns with a probability of about 80%, whereas *ser-2* mutants showed normal omega turns with only half of the probability (wild-type, 84%, *ser-2*, 55%)(Fig. 5h) [20]. We next investigated whether the *ser-2* mutation affects head bending by analyzing the head bending angle in the initiation of omega turns (Fig. 5c). We longitudinally divided worm images into 12 equal segments and calculated bending angles formed by three points (1–2-3) in the head region (Fig. 5d). We found that when *ser-2* mutant animals failed to close omega turns, they exhibited shallower head bends than wild-type animals (Fig. 5e).

To examine RME activity in the initial phase of omega turns, we next performed calcium imaging during omega turn behavior. We evoked the escape response by optogenetic stimulation of ASH and simultaneously imaged calcium signals from RME. Interestingly, in wild-type animals, RME had low calcium levels in the omega turn phase as well as in the reversal phase (Fig. 5f). This low calcium transient persisted until animals resumed forward locomotion. In contrast, *ser-2* mutants showed high calcium levels in RME neurons in both evoked reversals and omega turns. The ratio change in the initial phase of omega turns (Fig. 5f, black bars) in *ser-2* mutants was significantly higher than in wild-type animals (WT: 36.2 ± 4.1%, *n* = 7 from three animals; *ser-2*: 121.9 ± 10.3%, *n* = 7 from four animals; mean *R*_*omega turn*_/*R*_*forward*_ × 100 ± SEM%) (Fig. 5g). These results raise the possibility that high calcium levels in RME neurons are involved in shallow head bends in the initial phase of omega turns in *ser-2* mutants.

To further assess this possibility, we tested whether the shallow omega turn phenotype observed in *ser-2* mutants could be rescued by expressing wild-type SER-2 in RME neurons, again using the cell-specific rescue transgenes *Punc-25::ser-2(+)* and *Pser-2prom2::ser-2(+)* (Fig. 5h). We scored normal omega turns in response to anterior touches for each strain (*n* = 100). We observed that both rescue genes could restore normal omega turns in *ser-2* mutants with almost the same probability as wild-type animals, indicating that the SER-2 in RME neurons contributes to the execution of normal omega turns.

It has been suggested that SER-2 signaling in GABAergic VD body motor neurons facilitates ventral body muscle contraction and contributes to the execution of normal omega turns [20]. Therefore, we examined the rescue expression of SER-2 in GABAergic D-type neurons under the control of the *unc-30* promoter (*Punc-30::ser-2 (+)*), which can drive expression in the GABAergic DD and VD neurons, but not in the GABAergic non D-type neurons, including RME neurons [51]. We found that the D-type specific transgene (*Punc-30::ser-2 (+)*) provided a weak rescue activity; one of the two rescue lines moderately restored execution of normal omega turns, but the other line did not (Fig. 5h). These results suggest that SER-2 expression in RME neurons is sufficient for the execution of the complete omega turn, and that SER-2 expression in D-type motor neurons also contribute to it. The partial rescue with *Punc-30::ser-2(+)* indicates that ectopic expression of SER-2 in DD motor neurons, which may have an enhancing effect on contraction of dorsal body wall muscles, might hinder the ventral turns. Together, these results indicate that SER-2 acts in both head and body motor neurons to control omega turns.

## Discussion

Tyramine is known to control many behaviors in invertebrates, but how tyraminergic modulation can reconfigure an anatomically defined neural network to alter behavioral output is not fully understood. In the present study, we demonstrated that intracellular calcium levels of GABAergic RME neurons were downregulated by endogenous tyramine through the tyramine GPCR SER-2 during the backward moving states. Conversely, tyraminergic RIM neurons were activated during backward locomotion. RIM neurons are coupled by gap junctions to AVA neurons [18], which are the primary command interneurons that drive backward locomotion. The AVA neurons directly synapse on the downstream cholinergic motor neurons for facilitating backward movement, and have been shown to be active during backward locomotion [52]. Thus, we speculate that AVA activate RIM through gap junctions upon directional switching from forward to backward locomotion and that tyramine released from RIM consequently inhibits RME calcium activity via SER-2.

Although our data suggest a functional coupling between RIM and RME, RIM do not directly synapse onto RME [18]. Since RIM and RME are anatomically close together and, moreover, no tyramine reuptake transporter has been identified in *C. elegans*, tyramine spillover from RIM might activate SER-2 receptors on RME. In addition, gap junctions between RME neurons [52] might contribute to synchronize their activity. It has been shown that GABAergic VD body motor neurons are extrasynaptically regulated by tyraminergic signal through SER-2 [20]. Thus, tyramine appears to globally control head and body motor circuits in a paracrine manner, contributing to the execution of normal omega turns in *C. elegans*. It should be noted that unlike RME, VD, which lie throughout the body along the ventral nerve cord, are located far from RIM. In the case of VD, therefore, tyramine might diffuse through the pseudocoelom and, therefore, it might take time to respond. We speculate that tyramine regulates head bends and body bends on different time scales during omega turns.

**Fig. 6:**
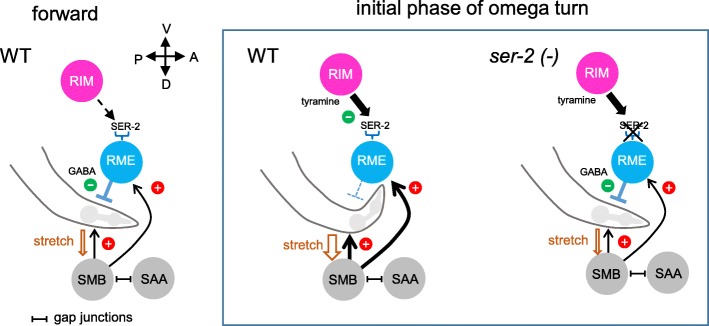
A model for regulation of head movements via tyraminergic modulation of RME neurons. Left: Schematic representations showing neural circuits that restrict head bending amplitude during forward movements in wild-type (WT) *C. elegans*. When animals bend their heads, the movement activates putative stretch receptors in the SMB and SAA neurons on the opposite side of the bend. The SMB neurons activate the ipsilateral muscles and RME neurons, which relax the contralateral muscles of the head and restrict the bending. Middle: In the initial phase of omega turns, tyramine released from the RIM neurons overrides the RME-mediated downregulation system through SER-2 and alleviates the proprioceptive feedback, so that the animals show exaggerated head bending. Right: In *ser-2* mutants, tyramine fails to induce the RME-mediated disinhibition of the head muscles, resulting in a shallow head bending in the omega turns

During forward locomotion, in which animals exhibit foraging behavior, head movement is properly suppressed by proprioceptive feedback not to make exaggerated head deflection. The SAA and SMB neurons, which extend long undifferentiated processes along the head, provide proprioceptive information regarding head position [18]. RME are thought to be involved in the proprioceptive feedback because RME anatomically receive ipsilateral inputs from these neurons and send outputs to the contralateral head muscles [52, 53]. A recent report showed that the amplitude of head bending can also be extrasynaptically regulated by inhibitory feedback between GABAergic RME and cholinergic SMD neurons [54]. The SMD is a group of cholinergic motor neurons that regulate head movement. When animals make deep turns, such as omega turns, it is desirable that the proprioceptive feedback and the extrasynaptic regulation through RME are inhibited. Indeed, RME-ablated animals exhibit highly exaggerated head deflection called the “loopy” phenotype [55]. In this study, we found that the tyramine system inhibited RME activity during backward movement and omega turns. This inhibitory tyraminergic modulation of RME is purposeful to alleviate the negative feedback regulation and to allow the deep head bends to make deep turns (Fig. 6).

It has been reported that during the touch-induced backward movement, tyramine hyperpolarizes the anterior muscles through activation of the anion channel-type receptor, LGC-55, thus relaxing the muscle and inhibiting head oscillation [20, 47]. Additionally, LGC-55 is expressed in several cholinergic head motor neurons and appears to inhibit muscle activity. Since increased intracellular calcium concentration facilitates neurotransmitter release, the downregulation of calcium levels in RME neurons through SER-2 most likely induces the inhibition of GABA release, which in turn disinhibits the head muscles. Indeed, an electrophysiological study showed that tyramine inhibited GABA release onto the ventral body muscles in a SER-2-dependent manner [20]. Thus, we speculate that the head muscles are regulated by tyramine through dual mechanisms, one through SER-2 in GABAergic RME motor neurons, and the other through LGC-55 in the cholinergic motor neurons and muscles, which appear to exert opposite effects on muscle activity; GABA modulation may limit the excessive hyperpolarization of head muscles during backward movement. Furthermore, disinhibition of the head muscles via RME neurons, together with disinhibition of the body wall muscles via downregulation of the D-type motor neurons, appears to participate in the execution of the complete omega turn.

How does tyraminergic signaling via SER-2 induce calcium decrease in RME? SER-2 is structurally conserved with invertebrate Gαi/o-coupled tyramine receptors [48] and has been suggested to couple to GOA-1, the *C. elegans* ortholog of the neural G-protein-alpha subunit of the Gαo class [56, 57]. The GOA-1 pathway has been shown to inhibit neurotransmitter release by antagonizing the EGL-30/Gαq pathway [58, 59]. Recent studies have shown that the GOA-1 pathway also affects inward rectifying potassium channels and mediates peptidergic inhibition of serotonin neurons [60]. In addition, mammalian Gα_o_ signaling has been implicated in inwardly rectifying potassium channels or voltage-gated calcium channels to inhibit neural activity [61, 62]. These intracellular mechanisms might also function in RME. Research combining in vivo calcium imaging, genetics, and behavioral analysis will elucidate how tyramine-induced G-protein signaling modulates intracellular calcium dynamics and behavior. These approaches would help provide further understanding of the mechanism of neuromodulation and its role in the flexibility of neural circuits in invertebrates and vertebrates.

## Conclusion

We found that intracellular calcium dynamics in the GABAergic head motor neurons is extrasynaptically downregulated by endogenous tyramine via the metabotropic tyramine receptor SER-2 during backward locomotion and omega turns. Our data suggest that this inhibitory tyraminergic modulation allows the animal to make the deep head bends and execute deep turns in escape behavior. We conclude that the findings of this study will contribute to further understanding of the mechanism of neuromodulation and its role in the flexibility of neural circuits.

## Additional file

Additional file 1: **Figure S1.** ICaST system for optogenetic control and simultaneous calcium imaging in freely moving animals. The light paths are indicated by colored arrows. Details are described in the Materials and Methods and a previous report [27]. **Figure S2.** R-CaMP2 imaging in RME. (**A**) Representative images of a transgenic animal expressing both R-CaMP2 and EGFP in RME neurons in forward (top panels) and backward (bottom panels) movements. Transmitted-light images (TD), raw fluorescent images of R-CaMP2 and EGFP, fluorescent merged images, and pseudocolor ratio images (R-CaMP2/EGFP) are shown. (**B**) Fluorescent intensity ratio values (*R*=R-CaMP2/EGFP) of RME in a freely moving animal are plotted as a function of time. (**C**) Quantitative analysis of mean fluorescent ratio changes of RME during forward (gray) and backward (red) locomotion. The mean value of R during forward locomotion was normalized as 100%. **Figure S3.** *tbh-1* mutants exhibit normal calcium responses in RME during backward locomotion. A representative calcium trace of RME in *tbh-1(ok1193)* mutants. **Figure S4.** *tph-1* and *cat-2* mutants exhibit normal calcium responses in RME during backward locomotion. (**A**) Biosynthetic pathways of serotonin and dopamine. Genes encoding synthetic enzymes are shown under the arrows. (**B**, **C**) Calcium dynamics of RME in* tph-1 (mg280)* (B) and *cat-2* (*jq6*) (**C**) mutants during spontaneous locomotion. **Table S1.** *C. elegans* strains used in this study. **Table S2.** Transgenic lines generated in this study. **Table S3**. Mutations and primers for genotyping. **Table S4.** Primers for molecular biology. (PDF 359 kb)
