# Supplementary material for: Role of tyramine in calcium dynamics of GABAergic neurons and escape behavior in Caenorhabditis elegans
Source: Zoological Lett. 2018 Jul 26;4:19. doi: 10.1186/s40851-018-0103-1 (PMC6062986; doi:10.1186/s40851-018-0103-1)
Supplement: Supplementary file 1 — Figure S1. ICaST system for optogenetic control and simultaneous calcium imaging in freely moving animals. The light paths are indicated by colored arrows. Details are described in the Materials and Methods and a previous report [27]. Figure S2. R-CaMP2 imaging in RME. (A) Representative images of a transgenic animal expressing both R-CaMP2 and EGFP in RME neurons in forward (top panels) and backward (bottom panels) movements. Transmitted-light images (TD), raw fluorescent images of R-CaMP2 and EGFP, fluorescent merged images, and pseudocolor ratio images (R-CaMP2/EGFP) are shown. (B) Fluorescent intensity ratio values (R=R-CaMP2/EGFP) of RME in a freely moving animal are plotted as a function of time. (C) Quantitative analysis of mean fluorescent ratio changes of RME during forward (gray) and backward (red) locomotion. The mean value of R during forward locomotion was normalized as 100%. Figure S3. tbh-1 mutants exhibit normal calcium responses in RME during backward locomotion. A representative calcium trace of RME in tbh-1(ok1193) mutants. Figure S4. tph-1 and cat-2 mutants exhibit normal calcium responses in RME during backward locomotion. (A) Biosynthetic pathways of serotonin and dopamine. Genes encoding synthetic enzymes are shown under the arrows. (B, C) Calcium dynamics of RME in tph-1 (mg280) (B) and cat-2 (jq6) (C) mutants during spontaneous locomotion. Table S1. C. elegans strains used in this study. Table S2. Transgenic lines generated in this study. Table S3. Mutations and primers for genotyping. Table S4. Primers for molecular biology. (PDF 359 kb) [file 40851_2018_103_MOESM1_ESM.pdf]

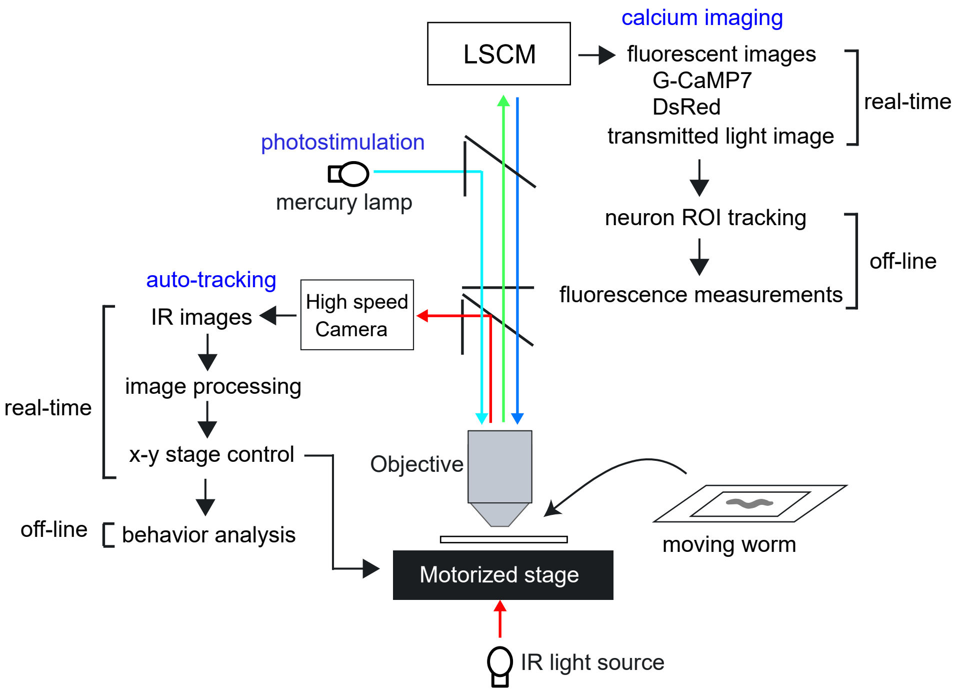


IR: infrared
LSCM: laser-scanning confocal microscope

Kagawa-Nagamura et al
Figure S1

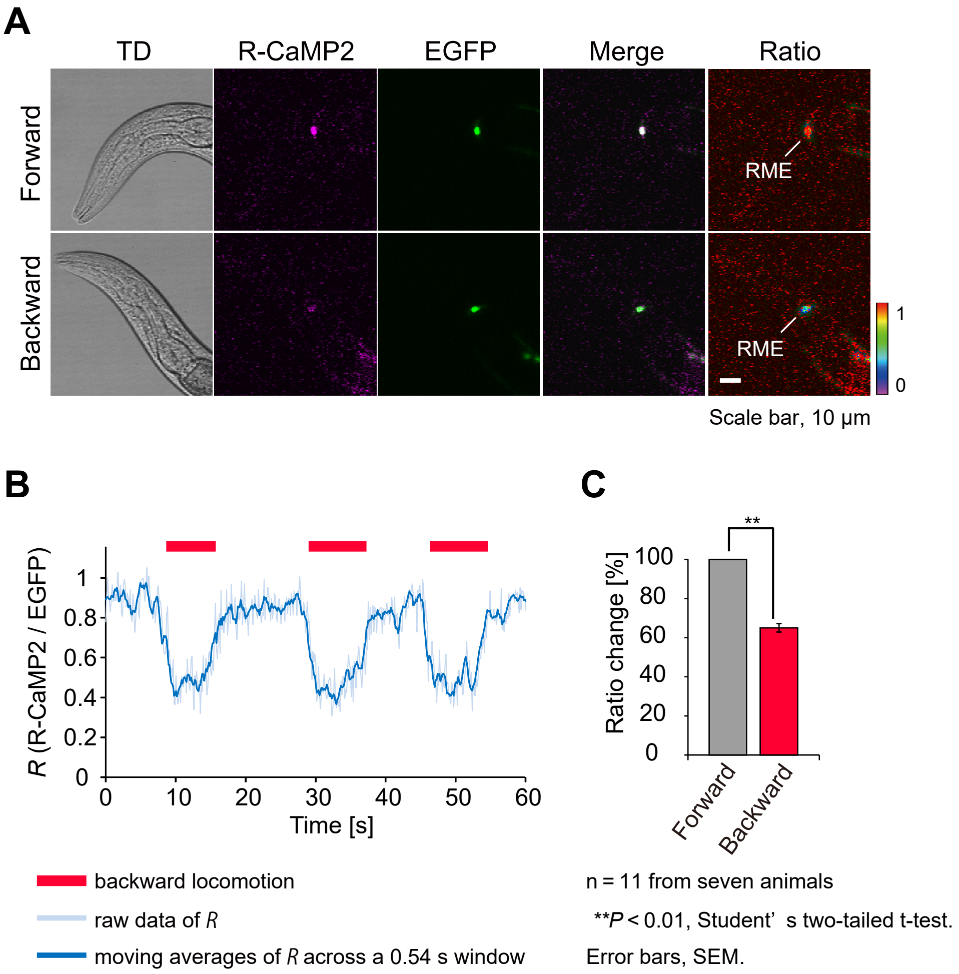


Kagawa-Nagamura et al
Figure S2

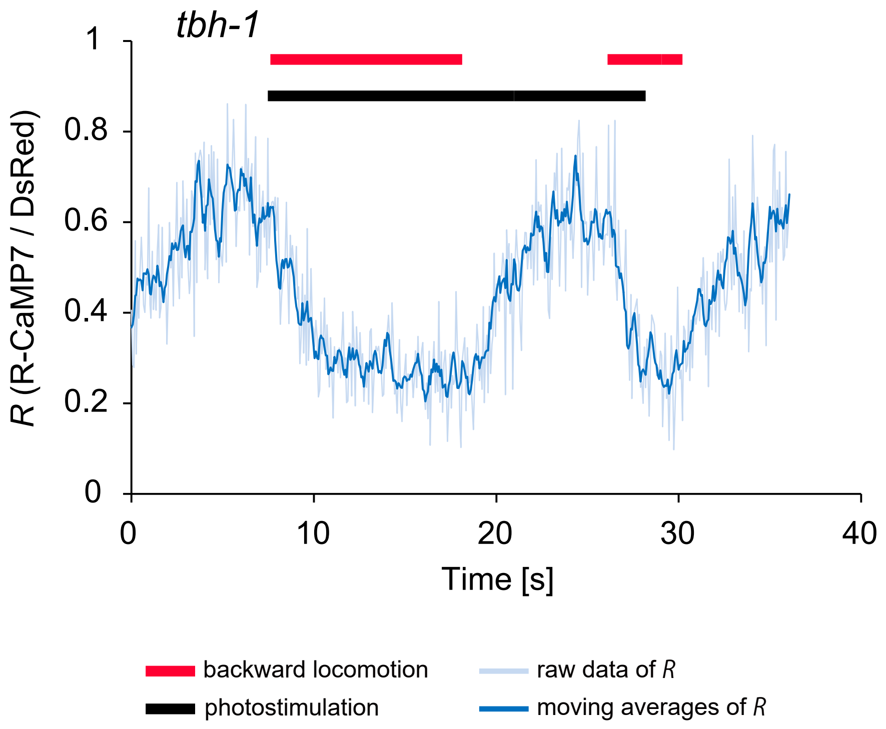


Kagawa-Nagamura et al

Figure S3

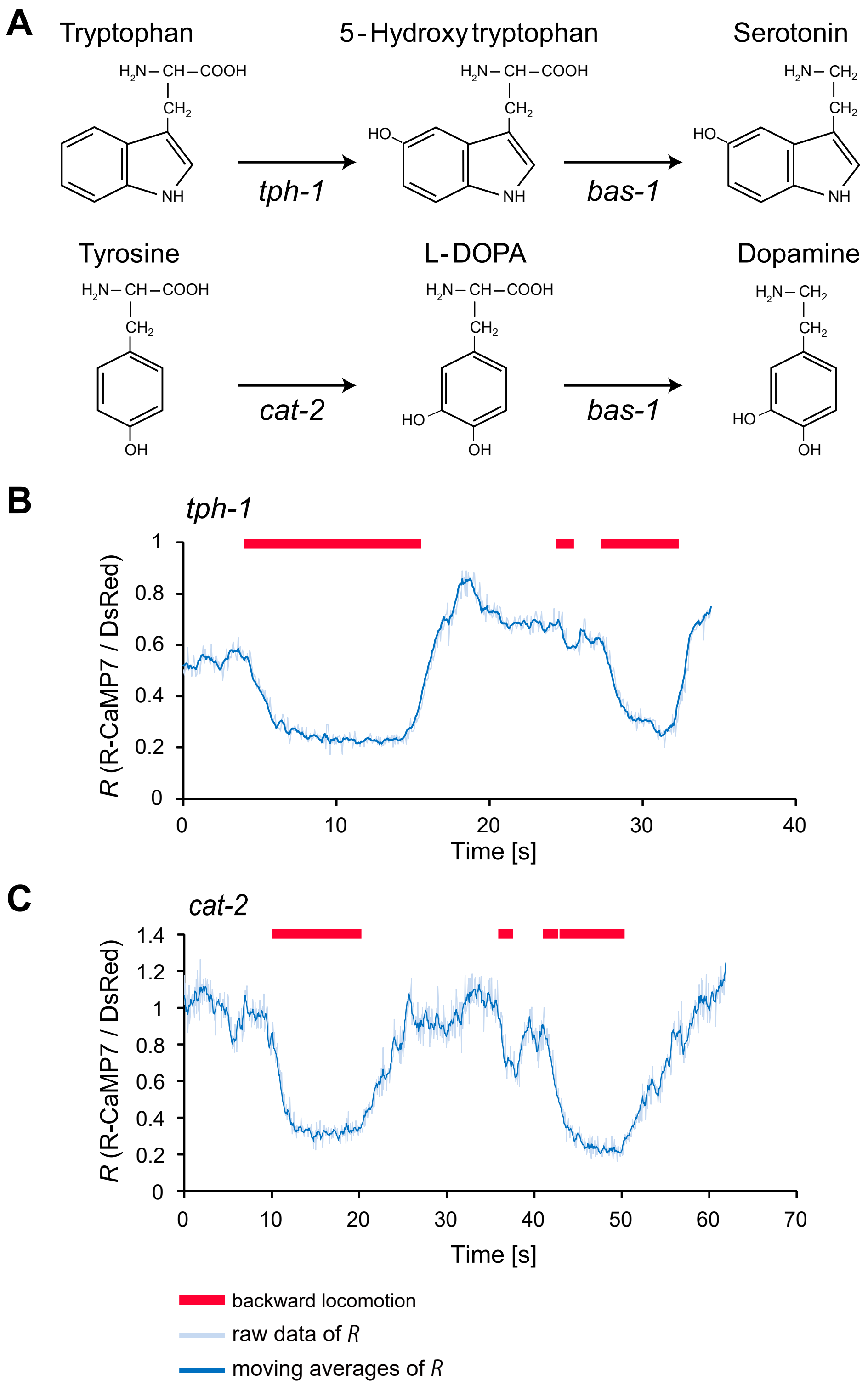


Kagawa-Nagamura et al
Figure S4

**Table S1 *C. elegans* strains used in this study**

| Strain | Genotype | Reference or Source |
|---|---|---|
| N2 | wild type | Brenner, Genetics, 1974 |
| RB993 | *tdc-1 (ok914) II* | The C. elegans Deletion Mutant Consortium, G3, 2012. OMRF[a] |
| RB1161 | *tbh-1 (ok1196) X* | The C. elegans Deletion Mutant Consortium, G3, 2012. OMRF[a] |
| RB1690 | *ser-2 (ok2103) X* | The C. elegans Deletion Mutant Consortium, G3, 2012. OMRF[a] |
| VC125 | *tyra-3 (ok325) X* | The C. elegans Deletion Mutant Consortium, G3, 2012. OMRF[a] |
| GR1321 | *tph-1 (mg280) cam-1(vs166) II* | Sze JY et al., Nature, 2000 |
| MT14680 | *lgc-55 (n4331) V* | Ringstad N et al., Science, 2009 |
| QJ6 | *cat-2 (jq6) II* | Usami A, Ph.D. Thesis, 2012 |
| QJ3063 | *jqIs6* | This study |
| QJ3064 | *jqIs71* | This study |
| QJ3065 | *jqIs72* | This study |
| QJ3067 | *jqIs75* | This study |
| QJ1412 | *jqEx439* | This study |
| QJ4110 | *tph-1 (mg280) II; jqIs6* | This study |
| QJ4111 | *cat-2 (jq6) II ; jqIs6* | This study |
| QJ1359 | *ser-2 (ok2103) X ; jqEx386* | This study |
| QJ1501 | *ser-2 (ok2103) X ; jqEx528* | This study |
| QJ1502 | *ser-2 (ok2103) X ; jqEx529* | This study |
| QJ1526 | *ser-2 (ok2103) X ; jqEx553* | This study |
| QJ1543 | *ser-2 (ok2103) X ; jqEx570* | This study |
| QJ1544 | *ser-2 (ok2103) X ; jqEx571* | This study |
| QJ4113 | *jqIs73 ; jqIs75* | This study |
| QJ4114 | *jqIs6 ; jqEx318* | This study |
| QJ1489 | *jqIs75 ; jqEx516* | This study |
| QJ4116 | *tdc-1 (ok914) II ; jqIs6 ; jqEx318* | This study |
| QJ4117 | *tbh-1 (ok1196) X ; jqIs6 ; jqEx318* | This study |
| QJ4118 | *ser-2 (ok2103) X ; jqIs6 ; jqEx318* | This study |
| QJ4119 | *lgc-55 (n4331) V ; jqIs6 ; jqEx318* | This study |
| QJ4120 | *tyra-3 (ok325) X ; jqIs6 ; jqEx318* | This study |
| QJ4122 | *ser-2 (ok2103) X ; jqIs6 ; jqEx386* | This study |
| QJ4123 | *ser-2 (ok2103) X ; jqIs6 ; jqEx422* | This study |
| QJ4112 | *ser-2 (ok2103) X ; jqIs6 ; jqEx528* | This study |

[a]OMRF: Oklahoma Medical Research Foundation, The *C. elegans* Deletion Mutant Consortium

**Table S2 Transgenic lines generated in this study**

| Transgenic array | DNA injected |
| --- | --- |
| *jqIs6* | *Punc-25::G-CaMP7* (100 ng/μl)<br>*Punc-25::DsRedx* (50 ng/μl) |
| *jqIs71* | *Punc-25::G-CaMP7 (ce)* (50 ng/μl)<br>*Punc-25::DsRedx* (50 ng/μl)<br>*pBluescript II SK+* (100 ng/μl) |
| *jqIs72* | *Punc-25::G-CaMP7(ce)* (50 ng/μl)<br>*Punc-25::DsRedx* (50 ng/μl)<br>*pBluescript II SK+* (100 ng/μl) |
| *jqIs73* | *Punc-25::G-CaMP7(ce)* (50 ng/μl)<br>*Punc-25::DsRedx* (50 ng/μl)<br>*pBluescript II SK+* (100 ng/μl) |
| *jqIs75* | *Ptdc-1::G-CaMP7(ce)* (50 ng/μl)<br>*Ptdc-1::DsRedx* (50 ng/μl)<br>*Ptbh-1::EBFP2* (50 ng/μl)<br>*pBluescript II SK+* (50 ng/μl) |
| *jqEx318* | *Psra-6::ChR2::mCherry* (100 ng/μl)<br>*Punc-122::mCherry* (50 ng/μl)<br>*pBluescript II SK+* (50 ng/μl) |
| *jqEx386* | *Punc-25::ser-2 (+)* (20 ng/μl)<br>*Punc-122::EYFP* (50 ng/μl)<br>*pBluescript II SK+* (130 ng/μl) |
| *jqEx422* | *Punc-17::ser-2 (+)* (20 ng/μl)<br>*Punc-122::EYFP* (50 ng/μl)<br>*pBluescript II SK+* (130 ng/μl) |
| *jqEx439* | *Punc-25::R-CaMP2* (50 ng/μl)<br>*Punc-47::EGFP* (50 ng/μl)<br>*pBluescript* (50 ng/μl) |
| *jqEx516* | *Punc-47::EGFP* (50 ng/μl)<br>*pBluescript II SK+* (150 ng/μl) |
| *jqEx528* | *Pser-2prom2::ser-2 (+)* (20 ng/μl)<br>*Punc-122::EYFP* (50 ng/μl)<br>*pBluescript II SK+* (130 ng/μl) |
| *jqEx529* | *Pser-2prom2::ser-2 (+)* (20 ng/μl)<br>*Punc-122::EYFP* (50 ng/μl)<br>*pBluescript II SK+* (130 ng/μl) |
| *jqEx553* | *Punc-25::ser-2 (+)* (20 ng/μl)<br>*Punc-122::EYFP* (50 ng/μl)<br>*pBluescript II SK+* (110 ng/μl) |
| *jqEx570* | *Punc-30::ser-2 (+)* (20 ng/μl)<br>*Punc-122::EYFP* (50 ng/μl)<br>*pBluescript II SK+* (130 ng/μl) |
| *jqEx571* | *Punc-30::ser-2 (+)* (20 ng/μl)<br>*Punc-122::EYFP* (50 ng/μl)<br>*pBluescript II SK+* (130 ng/μl) |

**Table S3 Mutations and primers for genotyping**

| Genotype | Mutation | Primer name | Sequence |
|---|---|---|---|
| *cat-2 (jq6) II* | 320bp deletion + 9bp insertion | cat-2_jq6_forward | CTTCACACGACGTGCTCCTA |
| | | cat-2_jq6_reverse1 | GTTGTCTGGAAGACCCGAAA |
| | | cat-2_jq6_reverse2 | CTTGAACTCTAACGCCTGATC |
| *lgc-55 (n4331) V* | 1986bp deletion | lgc-55_n4331_forward | CGGTGGTCAGTGGATAATTTTGTGC |
| | | lgc-55_n4331_reverse1 | GCGATTTGTGAATTGCAGAGCC |
| | | lgc-55_n4331_reverse2 | CGTCATCTATGAATTCGACCGGTTG |
| *ser-2 (ok2103) X* | 2043bp deletion | ser-2_ok2103_forward1 | GTGATGGTACAAGCTTCACC |
| | | ser-2_ok2103_reverse1 | CTTCGTCATTTGTGGTTTCG |
| | | ser-2_ok2103_forward2 | CTCCATCATATTCTCCTGCC |
| *tbh-1 (ok1196) X* | 981bp deletion | tbh-1_ok1196_forward | GGAAAGGACAATGGCACGTTGA |
| | | tbh-1_ok1196_reverse1 | AGGTGCTGAATTTTGCCTGCC |
| | | tbh-1_ok1196_reverse2 | CGAAGGTCCAACTGATCAGCCAA |
| *tdc-1 (ok914) II* | 629bp deletion | tdc-1_ok914_forward | GCTATGGCTTCATGTTGATGCTGC |
| | | tdc-1_ok914_reverse1 | GCCGTTATCAAAGGCGGAAACT |
| | | tdc-1_ok914_reverse2 | GCACATACACAGAAGCGGATCAC |
| *tph-1 (mg280) II* | 1305bp deletion | tph-1_mg280_forward | GCAAAGACCCCTCTCAACCTCATT |
| | | tph-1_mg280_reverse1 | GGAAACCATTCAGAACCGGTTGTC |
| | | tph-1_mg280_reverse2 | GCATGATGGCGAACGTATTGAGTG |
| *tyra-3 (ok325) X* | 781bp deletion | tyra-3_ok325_forward1 | GTTCTGAAACAGTTCACGGG |
| | | tyra-3_ok325_forward2 | GATTGCCTCATGAGAGCTTC |
| | | tyra-3_ok325_reverse1 | GGAGAAGAGTCCGTTTATCG |

**Table S4 Primers for molecular biology**

| No. | primer name | Sequence | Comments |
|---|---|---|---|
| 1 | G-CaMP7(CE)_NheI#F1 | CTTACTAGTGCTAGCATGAGAGGAAGTCATCATCA | 1 and 2 were used to amplify the 1.4 kb codon-optimized G-CaMP7 cDNA for *C. elegans* |
| 2 | G-CaMP7(CE)_NheI#R1 | TTTTTTTTGCTAGCTTATTTGGCAGTCATCATCT | |
| 3 | R-CaMP2_Bgl#R1 | TTTTAGATCTCTATGGGTTGGACTCCACGT | 3 and 4 were used to amplify the 1.5 kb R-CaMP2 cDNA |
| 4 | R-CaMP2_Not#F1 | CTTACTAGTGCGGCCGCATGGGTTCTCATCATCATCATC | |
| 5 | DsRedx_NheI#F1 | CTTACTAGTGCTAGCATGGCCTCCTCCGAGGACGT | 5 and 6 were used to amplify the 0.68 kb DsRed-Express-1 cDNA |
| 6 | DsRedx_NheI#R1 | TTTTTTTTGCTAGCCTACAGGAACAGGTGGTGGC | |
| 7 | ser-2 cDNA Bgl#R1 | TTTTAGATCTCTAAGGTTGCGCACTCATTC | 7 and 8 were used to amplify the 1.3 kb full-length *ser-2* cDNA |
| 8 | ser-2 cDNA Not#F1 | CTTACTAGTGCGGCCGCATGTTCCGAAATTACACTGA | |
| 9 | ser-2prom2#F1 | ATTGGCTGCAGATTTTATGACTTTCACTAGAAATG | 9 and 10 were used to amplify the 4.7 kb *ser-2prom2* promoter |
| 10 | ser-2prom2#R1 | AGCTGGATCCTTTTGCAAATTACTTGAGGCTG | |
| 11 | tbh-1 promoter#F1_Bam | AGCTGGATCCCCTTATGTATCTCATTGCTATTT | 11 and 12 were used to amplify the 2.8 kb *tbh-1* promoter |
| 12 | tbh-1 promoter#R1_Not | CTTACTAGTGCGGCCGCTTTCTGAAATCGTATAGTAATGG | |
| 13 | tdc-1p_BamHI#R1 | AGCTGGATCCTTGGGCGGTCCTGAAAAATG | 13 and 14 were used to amplify the 4.4 kb *tdc-1* promoter |
| 14 | tdc-1p_HindIII#F1 | TTTTTTTTAAGCTTAAGGGAGAGAGTATTGCAGTGG | |
| 15 | unc-25p_BamHI#R1 | AGCTGGATCCTCTTTTTTGGCGGTGAACTG | 15 and 16 were used to amplify the 1.9 kb *unc-25* promoter |
| 16 | unc-25p_HindIII#F1 | TTTTTTTTAAGCTTTGTGCATGCAAAAAACACCC | |
| 17 | unc-30_SphI#F1 | TTTTTTTTGCATGCTCGATGACACTTTTTGATGTGT | 17 and 18 were used to amplify the 2.4 kb *unc-30* promoter |
| 18 | unc-30_BamHI#R1 | AGCTGGATCCGTGTTGATCTTTTGCCGGAG | |
| 19 | unc-54 3'#R1 | GACTTAGAAGTCAGAGGCAC | 19-25 were used to verify the sequences of DNA constructs |
| 20 | unc-86_UTR#R1 | CAAAGCGAATTGCTAAACCC | |
| 21 | pFX#F1 | GCAAGGCGATTAAGTTGGGT | |
| 22 | pPD49.26#F1 | CGTTGGCCGATTCATTAATG | |
| 23 | ser-2#R2 | GTAGACAACCCGCAACTATC | |
| 24 | ser-2 cDNA #F2 | CATTGGGATTATAGTCACGC | |
| 25 | unc-25p#F1 | CGGAATAGGGAAAAGCTCAG | |
